# Using a brain-machine interface to control a hybrid upper limb exoskeleton during rehabilitation of patients with neurological conditions

**DOI:** 10.1186/s12984-015-0082-9

**Published:** 2015-10-17

**Authors:** Enrique Hortal, Daniel Planelles, Francisco Resquin, José M. Climent, José M. Azorín, José L. Pons

**Affiliations:** Brain-Machine Interface Systems Lab, Miguel Hernández University of Elche, Av. de la Universidad, S/N, Elche, 03202 Spain; Rehabilitation Group, Cajal Institute, Spanish National Research Council, Madrid, Spain; Department of Physical Medicine and Rehabilitation, Hospital General Universitario de Alicante, Alicante, Spain

**Keywords:** BMI, EEG, Rehabilitation, Neurological condition, Exoskeleton, Functional electrical stimulation, Motor imagery, Arm movement intention detection

## Abstract

**Background:**

As a consequence of the increase of cerebro-vascular accidents, the number of people suffering from motor disabilities is raising. Exoskeletons, Functional Electrical Stimulation (FES) devices and Brain-Machine Interfaces (BMIs) could be combined for rehabilitation purposes in order to improve therapy outcomes.

**Methods:**

In this work, a system based on a hybrid upper limb exoskeleton is used for neurological rehabilitation. Reaching movements are supported by the passive exoskeleton ArmeoSpring and FES. The movement execution is triggered by an EEG-based BMI. The BMI uses two different methods to interact with the exoskeleton from the user’s brain activity. The first method relies on motor imagery tasks classification, whilst the second one is based on movement intention detection.

**Results:**

Three healthy users and five patients with neurological conditions participated in the experiments to verify the usability of the system. Using the BMI based on motor imagery, healthy volunteers obtained an average accuracy of 82.9 ± 14.5 %, and patients obtained an accuracy of 65.3 ± 9.0 %, with a low False Positives rate (FP) (19.2 ± 10.4 % and 15.0 ± 8.4 %, respectively). On the other hand, by using the BMI based on detecting the arm movement intention, the average accuracy was 76.7 ± 13.2 % for healthy users and 71.6 ± 15.8 % for patients, with 28.7 ± 19.9 % and 21.2 ± 13.3 % of FP rate (healthy users and patients, respectively).

**Conclusions:**

The accuracy of the results shows that the combined use of a hybrid upper limb exoskeleton and a BMI could be used for rehabilitation therapies. The advantage of this system is that the user is an active part of the rehabilitation procedure. The next step will be to verify what are the clinical benefits for the patients using this new rehabilitation procedure.

## Background

Currently, the number of people suffering from motor disabilities or reduced mobility is increasing. Cerebro-Vascular Accidents (CVAs), i.e. strokes, are ones of the main causes of these problems. The number of people with probability of suffering a CVA is growing worldwide mainly due to the aging population [1]. This value is expected to reach in 2030 an increase of 24.9 % compared to 2010 levels [2]. According to the Spanish Society of Neurology, the number of stroke patients at Spanish hospitals has increased by 40 % over the last 15 years [3]. As reported by the World Health Organization (WHO), 15 million people suffer stroke worldwide each year, and around 5 million of them are permanently disabled [4]. All these facts evidence the necessity of improving not only prevention mechanisms but also rehabilitation procedures for people with these conditions.

Due to certain shortcomings of conventional therapy, rehabilitation systems applied after a CVA have experimented an important improvement in recent years. After conventional therapies, motor impairments as paralysis persist in a large percentage of stroke population. Recovery of motor skills is commonly very low after stroke [5] and, compared to lower limb, improvements of upper limb motor function are even lower [6]. By these facts, novel rehabilitation approach, as robot-aided rehabilitation and functional electrical stimulation (FES) were introduced, with the aim to improve effectiveness of therapy.

Several publications have showed improvements in upper limb motor function after rehabilitation therapies based on robotic devices [7, 8] and FES [9, 10]. Furthermore, the combined use of both technologies has shown promising results in terms of motor recovery after stroke [11, 12]. The main advantage of using the hybrid approach is that, individual limitations are overcome, generating in this way a more robust concept [13]. Robotic devices generally apply external mechanical forces to drive joint movements, while FES-based therapy facilitates exercise execution leaded by the participant’s own muscles. This last approach yields several benefits considering motor recovery, such as muscle strength [14] and cortical excitability [15]. Further, even when stroke participant does not contribute to voluntary movement these advantages are still present. However, the use of FES elicits the fast occurrence of muscle fatigue due to non-physiological recruitment (unnatural) of the motor units. Muscle fatigue decreases the efficacy of therapy and also entails other drawbacks, that is why, effort are always targeted to prolong the appearance of its effects. Moreover, the nonlinear and time variant behavior of the muscles during FES generate a less accurate motor control response. This problem can be addressed by using an exoskeleton, in order to cooperatively aid the movements. The inclusion of robotic device avoids stimulate arm’s muscles to overcome gravity effects, and hence, release the system from patients discomfort generated when arm muscles are constantly stimulated for this purpose. So, the main idea begins the hybrid approach based on reaching movement rehabilitation is that the exoskeleton compensate again gravity and FES assists the patient for movements execution.

Besides physical rehabilitation [16], an important question arises from the neurological level due to the neuroplasticity [17]. In this regard, multiple works focused on this kind of rehabilitation are being developed [18–20]. Brain-Machine Interfaces (BMIs) are conceived as a powerful tool for rehabilitation of CVA patients. By using these interfaces, patients are an active part of the process because the control commands are generated directly from their brain activity. Thus, not only would the rehabilitation improve from the physical point of view, but also from the neurological perspective [21]. With this system, patients are actively involved in their rehabilitation process.

To achieve a greater involvement of the patients, the use of a BMI can represent an important improvement. Several studies based on BMIs have demonstrated that people with disabilities are able to control properly systems such as a wheelchair [22], robots [23] or other devices such as a PC mouse [24] or a web browser [25]. The main objective in these works was to provide a new way to interact with the environment and facilitate daily life activities. However, these systems were not designed to restore the affected capacities of the users. Other works used brain signals to command systems that provide aid in physical and neurological rehabilitation as in [26].

Thanks to neuroscience, it is well known that many brain cognitive processes are located around the cortex. When BMIs are used in motor rehabilitation, parietal and frontal lobes are more interesting than others because they take part in intention, planning and decision of making a movement [27]. Therefore, signals acquired from these lobes can provide more information about the will to imagine or perform a movement. By using their brain signals, patients in rehabilitation could command a device to provide them some voluntary mobility. It is demonstrated that a FES therapy triggered by Electromyography (EMG) has advantages as it integrates the concept of sensorimotor feedback [9]. Using electroencephalography (EEG), follows the same approach, FES simulates normal operation of neural connections, taking the cortical level signals instead of peripheral signals (EMG) to trigger the execution of the task.

In this paper, a BMI allows, through two different methods, the control of a hybrid upper limb exoskeleton. Both methods are based in the analysis of EEG signals. EEG techniques are a non-invasive method which provides a higher patient acceptance, eliminates the health risks of operations and reduces impediments related to ethical issues. The exoskeleton is used to assist the upper limb rehabilitation process by performing extension and flexion elbow movements of the arm applying FES. The methods used in the BMI are based on motor imagery and movement intention detection through the Event-Related Desynchronization (ERD) and Event-Related Synchronization (ERS) detection. The accuracy of both methods are analyzed to demonstrate their usability and to determine which of them is better to be used in the rehabilitation therapy.

## Methods

### Participants

Three healthy volunteers (H1-H3) and five patients (P1-P5) were recruited to the study. None of the healthy subjects reported any type of neurological and psychiatric disorders. All of them were men, aged between 25 and 29 (27.3 ± 2.1). Only one user (H3) was left-handed. The group of patients was composed of one male (P5) and four females, with ages between 29 and 59 (45.2 ± 11.3). Two of them were left-handed (P1 and P3). In relation to their neurological conditions, P2 and P3 had suffered a stroke with right hemiplegia, P1 and P4 had left hemiplegia, and P5 suffered from spastic quadriplegia. The complete patients’ demography is indicated in Table 1. Upper limb motor dysfunction was evaluated based on the scale presented in [28]. This scale relies on three tests, listed below:

1. Pinch grip: 2.5 cm cube between thumb and forefinger.

**Table 1:** Patient’s demographics

Patient	Gender	Date of birth	Diagnosis	Time from injury	Spasticity	Motricity index
						(April 2014)
P1	Female	19/08/1984	Ischemic stroke (carotid dissection)	3 years	Left hemiparesis	Grip: 11
						Elbow: 19
						Shoulder: 14
						ARM SCORE: 44
P2	Female	24/09/1963	Ischemic stroke (carotid dissection)	3 years	Right hemiparesis	Grip: 0
						Elbow: 14
						Shoulder: 14
						ARM SCORE: 28
P3	Female	29/04/1955	Ischemic stroke (trombosis)	1 year	Right hemiparesis	Grip: 11
						Elbow: 19
						Shoulder: 14
						ARM SCORE: 44
P4	Female	07/06/1966	Ischemic stroke (hereditary spherocytosis)	8 years	Left hemiparesis	Grip: 0
						Elbow: 14
						Shoulder: 14
						ARM SCORE: 28
P5	Male	01/10/1973	Traumatic brain injury (traffic accident)	11 years	Spastic quadriplegia	Grip: 11
						Elbow: 14
						Shoulder: 14
						ARM SCORE: 39
2. Elbow flexion: from 90°, voluntary contraction/movement.
3. Shoulder abduction: from against chest.

Test 1 was scored as follows:

- 0 = No movement
- 11 = Beginnings of prehension
- 19 = Grips cube but unable to hold against gravity
- 22 = Grips cube, held against gravity but not against weak pull
- 26 = Grips cube against pull but weaker than other/normal side
- 33 = Normal pinch grip

The following score was used for Test 2 and 3:

- 0 = No movement
- 9 = Palpable contraction in muscle but no movement
- 14 = Movement seen but not full range/not against gravity
- 19 = Full range against gravity, nor against resistance
- 25 = Movement against resistance but weaker than other side
- 33 = Normal power

Results are shown in the “Motor Index” column of Table 1. The patients enrolled were recruited from the University General Hospital of Alicante (Spain). The experimental procedures were approved by the Ethics Committee of the Miguel Hernandez University of Elche (Spain) and the University General Hospital of Alicante. All users (patients and healthy subjects) gave their consent to take part in the experiments.

### Experimental setup

**Fig. 1:**
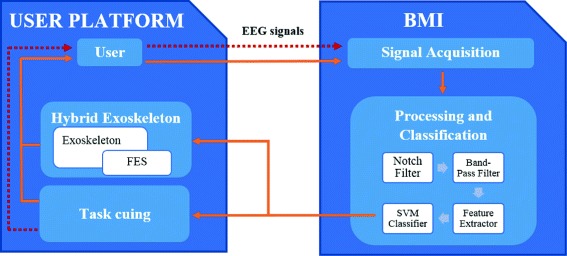
Experimental setup diagram. The diagram represents the offline and online setups. In the offline test (red dashed line), the Task cuing block guides the user and EEG signals are registered for further analysis. In the online test (orange solid line), the EEG information is processed and classified to control the elbow movements (using the FES in the arm supported by the exoskeleton)

The experiment consists in using a hybrid exoskeleton powered by FES and controlled by a BMI for the rehabilitation of the upper limb. The main goal was to trigger the task execution by mean of volitional cortical signals to mimic supraspinal connection existing in healthy subjects, and provide a positive sensorimotor feedback. The experimental procedure relied on carrying out elbow flexion/extension in the horizontal plane driven by the hybrid exoskeleton. The range of movement was adjusted at the beginning of the test according to each user capabilities. Two experiments based on different approaches have been used to control the hybrid exoskeleton from the brain activity: 1) using motor imagery, and 2) detecting the intention of moving the arm. The subject was sitting in front of a computer screen, in which the task cuing interface was shown. The experimental setup is shown in the schematic diagram represented in Fig. 1. The red dashed line represents the offline configuration which was used for both user and classifier training. The orange solid line includes the control of the arm movement through the FES applied in the online tests.

#### Brain-machine interface

The BMI implemented in this paper is similar to the interface used in previous works. In [29], the BMI allowed the control of a planar robot using two methods based on the differentiation of two mental tasks. This BMI is based on EEG.

EEG biosignals are acquired using the g.USBamp amplifier (g.Tec Medical Engineering GmbH, Austria). This amplifier has 16 channels and the signals are registered with a sampling frequency of 256 Hz using a 24 bits A/D converter. Raw signals were notch filtered (50 Hz) to eliminate the power line interference. The software used to register the EEG signals has been programmed in Matlab Development Environment (The Mathworks Inc., Natick MA) using the API (Application Programming Interface) provided by the manufacturer (gUSBamp MATLAB API). Both, signal processing and task cuing interface have also been developed using Matlab Development Environment. Signals were acquired through 16 active electrodes of g.LADYbird model (g.Tec Medical Engineering GmbH, Austria). These electrodes are composed of a sintered Ag/AgCl crown with a 2-pin safety connector, that make them less affected by motion artifacts, electromagnetic interferences and improve the signal-to-noise ratio in relation to the passive ones. Electrodes are placed using the cap g.GAMMAcap (g.Tec Medical Engineering GmbH, Austria), allowing a fast placement.

As the areas of the brain where the motor activity is better reflected are the parietal and frontal lobes, the electrodes were uniformly distributed in these regions of the scalp. Electrodes are located in the following positions (according to the International 10/10 System): Fz, FC5, FC1, FCz, FC2, FC6, C3, Cz, C4, CP5, CP1, CP2, CP6, P3, Pz and P4. The system used a mono-auricular reference placed on the right earlobe and the ground sensor is placed on the AFz position.

#### Hybrid upper limb exoskeleton

Stroke patients are usually unable to perform arm movement due the resistance to arm extension associated with overactivity of muscles generated by spasticity [30]. During the last decade have been reported evidences about the FES benefits for rehabilitation to reinforce ascending neuronal pathways by providing sensorial feedback [31]. This feedback is associated with cortical changes that can generate recovery of functional movement. However, FES must be applied under controlled environments in order to decrease the muscle fatigue onset and ensure safety. In this study, an ArmeoSpring exoskeleton (Hocoma AG, Switzerland) provides the arm support. By this combination the whole affected arm is supported by the mechanical structure avoiding stimulation of muscles to overcome gravity. In addition, shoulder and wrist joints are blocked, focusing exclusively on the elbow flexion/extension. This passive exoskeleton has been widely used for rehabilitation after stroke [32], spinal cord injury [33] and also sclerosis multiple [34].

Electrical stimulation was applied to the triceps and biceps muscles for elbow extension/flexion respectively, limited in the horizontal plane. The FES system consists of the electrical stimulator INTFES (Tecnalia Systems, S.L., Spain) and traditional surface electrodes (Pals Platinum - rectangle 5 × 5 cm).

Biphasic electrical pulses were delivered on targeted muscles at frequency of 40 Hz, pulse width of 350 *μ*s and amplitude modulated by a feedback controller. The maximum stimulation amplitude on biceps and triceps muscles was adjusted for each patient before session. This amplitude was found by gradually increasing the pulse amplitude leaving constant others parameters until the elbow flexion/extension movement response was generated within comfortable limits. This maximum value was incorporated in the feedback control as threshold values.

The reference trajectory was implemented using the Minimum Jerk function [35]. It was a smooth trajectory reference with bell-shape velocity used to model the human reaching profile. The human elbow position was estimated from the exoskeleton joints sensors, and a PID controller was implemented in order to determine the FES assistance level. The PID constant parameters were adjusted by Ziegler and Nichols method [36], using the average movements responses of healthy subjects.

### Experimental procedure - motor imagery

The first test has to be able to detect when volunteers are mentally performing a movement with the impaired arm. In this mental task, users have to image that they are grasping an object. According to Decety and Lindgren [37], the mental activity generated by a performed and imagined movement follows the same cortical pattern. Taking advantage of this statement, people with motor disabilities can control their arm movement execution by mean of a BMI system. A synchronous BMI is in charge of this detection. Furthermore, the use of the visual interface and the hybrid exoskeleton is designed to facilitate sensorimotor feedback, which is crucial to facilitate cortical reorganization and motor improvement.

#### Test protocol

Tests based on BMI motor imagery detection are divided into two phases. Firstly, an offline analysis is performed for both user training and classifier model obtainment. Then, real-time control of the volitive elbow movements (through the activation of the FES system) is performed. These tests were performed in a dedicated room where external stimuli did not disturb the user.

**Fig. 2:**
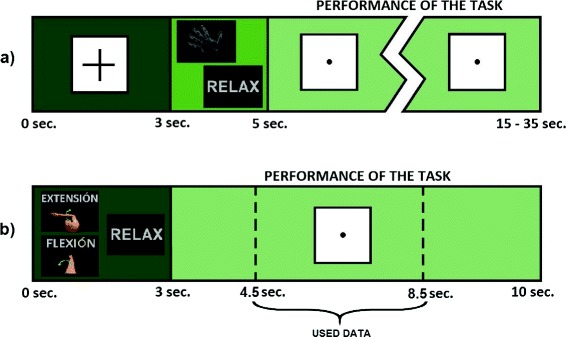
Training paradigms. **a** Task sequences of the motor imagery test. The graphical interface shows a cross during three seconds. Afterward, the task to be performed is shown during two seconds. Finally, 10 or 30 s are established to perform the demanded task (motor imagery or rest time respectively). **b** Task sequence of the movement intention test. Firstly, the corresponding task is shown during three seconds. After that, seven seconds are established to perform the task, where the data between the seconds 4.5 and 8.5 are used as valid data to the classifier

The offline phase relies on four runs applying a similar paradigm described in [38]. Figure 2a shows this approach that guides the user during the test. First, a cross is shown during three seconds. This cross represents the beginning of every cycle of imagery task and it is used as a break time for the user. Then, a representative image of the task to be performed (motor imagery task or rest state) is shown for two seconds. Lastly, a period of 10 or 30 s is established to perform the appropriate mental task (the motor or rest task, respectively). During the motor imagery period, the users must imagine grasping an object until the 10 s period is finished. This process is repeated four times per run for each task. A couple of minutes are established between runs as a rest for the users (if necessary). Hence, during this training phase a total of 160 s of motor imagery task and 480 s of rest state are obtained.

Depending on the user, the behavior of the system can be very variable. For this kind of experiments, the number of false detections during motor imagery task must be kept as low as possible (low False Positive rate). To this end, a model which tries to aid the correct detection of the rest state is designed. For this reason, there is an imbalance dataset depending on the class. The amount of data of rest state in proportion to the trials of motor task varies and it is selected individually according to the accuracy of the system for each volunteer (these accuracies are shown in section *Results and discussion*).

After this training, the created model of the classifier is tested by during the online test. In this test, the commands to control the hybrid exoskeleton are generated in accordance with the EEG online classification. This test includes four runs where the orthosis supports the arm against gravity and the users generate the commands to assist the elbow flexion/extension by FES. The movement performed is alternatively switched between “extension” and “flexion” depending on the current position of the arm (that is obtained from the exoskeleton joints sensors). All subjects had their arm initially flexed.

Each run of the online test includes 10 repetitions per task (motor imagery task and rest state). Rest state was always fixed to 10 s, whilst motor imagery state had 10 s duration only if this task was not detected correctly previously. A control command is generated only when three consecutive detections are identified during the period established to that end. This restriction avoids a high number of FPs, but adds a short delay.

Each volunteer performs four online runs in which the flexion/extension movements are generated. During these online tests, after each FES activation an extra period of five seconds was included, corresponding to the maximum time to reach the target position (less than two seconds was usually enough to complete the movement). Correct detections (True Positives) and erroneous detections (False Positives) of motor imagery task are computed and subsequently analyzed in order to evaluate the performance of the system.

#### Signal processing

The following signal processing steps were implemented to discriminate the rest state from imagined motor task using 16 EEG channels. Firstly, the data belonging to the performance of the tasks (when the screen is showing the dot) are segmented in windows of one second with 500 ms of overlapping. This way, 19 and 57 trials are obtained for each repetition (for the periods of 10 and 30 s, respectively). This data segmentation provides a total of 304 trials $\left (4 \: runs \cdot 4\: \frac {repetitions}{run} \cdot 19\: \frac {trials}{repetition}\right)$ of the motor imagery task and up to a maximum of 912 trials $\left (4\: runs\cdot 4\: \frac {repetitions}{run} \cdot 57\: \frac {trials}{repetition}\right)$ of the rest state.

In order to preserve the frequency components that provide more information related to motor imagery and to remove the DC component of the signals, a band pass filter (4th order Butterworth) is applied between 5 and 40 Hz [39, 40]. Acquired signal of each electrode is contaminated by the information of neighbor neurons, due the high population of neuron that are interconnected in the brain. As a consequence, a spatial filter can reduce the influence of other parts of the cerebral cortex by subtracting the information of near electrodes. In this work, a Laplacian algorithm is implemented and the subtraction is related to the distance between electrodes as follows:

(1) $$ Vi^{LAP}=Vi^{CR}-\sum_{j\epsilon Si}g_{ij}Vj^{CR}  $$

where *Vi*^*LAP*^ is the result of applying this algorithm to the electrode *i*, *Vi*^*CR*^ is the signal recorded at electrode *i* signal before the transformation and,

(2) $$ g_{ij}=\frac{\frac{1}{d_{ij}}}{\sum_{j\epsilon Si}^{}\frac{1}{d_{ij}}}  $$

where *Si* contains all the electrodes except electrode *i*, and *d*_*ij*_ is the distance between electrodes *i* and *j*.

Moreover, these signals are subsequently normalized regarding the variance in each processing window for all channels independently. Thus, the obtained signals are more stable over time.

Finally, the frequency features of the signals are calculated using the periodogram method [41]. This procedure allows the extraction of the frequency characteristics of the signals converting them from the time domain to the frequency domain. This procedure is a Power Spectral Density (PSD) estimation which uses the Discrete Fourier Transform (DFT). It is a biased estimator (even though the mean value of the periodogram will converge to the true PSD, the variance does not decrease to zero). The features taken into account for the classification are between 8 and 36 Hz every 1 Hz, selecting only the frequencies which provide a representative contribution of the mental activity. As a result, 29 features are obtained for each electrode. The signal processing allows getting a group of features that represent the mental task performed by the volunteers.

The selected features were studied in previous works to assess the possibility of reducing the number of electrodes or frequencies used in the final application [42, 43]. However, the best combination of electrodes and/or frequencies are very depending on the users, becoming impossible to generalize the reduction of features. On the other hand, the method applied in these experiments has been checked in previous works obtaining good results in healthy subjects (as in [23] and [29]).

#### Classification

The classifier used to distinguish between the mental tasks (i.e. between the rest state and the imagined motor movement) is based on Support Vector Machines (SVM). This kind of classifiers is commonly used in BCI data-sets [44, 45]. To perform the classification, SVM makes use of a hyperplane or groups of them in a very high (even infinite) dimensional space to distinguish the different classes. This classifier solves the optimization problem of maximizing the margin between hyperplanes by standard quadratic programming techniques [46]. The accuracy of the SVM-based classifier depends on the kernel used. The most widely used functions in the field of BMI are the Gaussian and the Radial Base Function (RBF) [44]. In this work, a SVM-based system with a RBF kernel is implemented, and an one-step multiclass strategy is selected.

In order to differentiate the tasks, the SVM-based system needs a personalized model created previously. Using this model, the system classifies the tasks which are being performed by the user. To avoid (or reduce) wrong classifications, a discrimination method was applied during online test. In this case, the output of the classifier is the motor imagery task only if, at least, three consecutive motor imagery tasks have been detected. Otherwise, the systems returns an uncertainty value and the systems remains in the rest state.

### Experimental procedure - movement intention

A BMI system is used in the second experiment to detect the intention of performing a flexion/extension movement. In this case, the BMI is based on the ERD phenomenon in order to detect this intention, as it was described in [47]. This cognitive process is described as a spectral power decrease in mu and beta frequency bands relative to a previous resting time. This brain behavior is produced by the intention and the performance of a movement. It starts before the movement actually begins, and ends, approximately, when the movement is finished. Moreover, after the ERD, an ERS occurs and the spectral power is increased and reestablished [48]. Considering that patients with motor impairment have difficulties to move, the activation of an external device on their will to support their movement execution could serve to gradually improve motor function and facilitate new neural connection (plasticity). This approach allows performing the elbow flexion/extension assisted by the same hybrid exoskeleton mentioned before.

#### Test protocol

These tests have been carried out under the same conditions presented previously to avoid external stimuli which can disturb the user. This fact is specially important in this kind of rhythmic sensory cues [49]. In this case, the BMI is based on the detection of elbow extension or flexion movement intention, even when the patient cannot perform it. Moreover, it should be able to detect when the subject is relaxed.

As with the motor imagery approach, this test is divided into two phases: the first one is dedicated to train the user and to adjust the classifier of the BMI, and the second one is focused on the real time tests. In both phases, subjects should try to move their arm only one time per each repetition dedicated to the motor task. However, they were warned not to react to any visual stimulus and to wait at least one second after each transition represented as a change of image on the screen, since it evokes potentials in the brain which could disturb the purpose of our experiment. Furthermore, if patients could perform the movement with residual arm functions, then we allowed them to carry it out without additional aid. Healthy users were asked to move the arm only a bit, emulating that they had difficulties to perform the movement. At the end of each motor task, they had to end the movement to be prepared for the next movement.

The experiment starts with six runs following the paradigm described in Fig. 2b. Each run is composed of five extension and flexion movements interspersed with ten periods of relax between each movement intention. A computer screen is used to guide the subjects through the different steps. Each task lasts ten seconds: the first three seconds are used to show the task to be performed (relax, extension or flexion) with a representative image; and then seven seconds are established to carry it out (in the meantime, a dot in the middle of the screen is shown). In order to adjust the classifier, only four seconds in the middle of the last six seconds are used (labeled as “Used data” in Fig. 2b). The remaining data are discarded because the relevant information is around two seconds before the movement intention and the execution of the attempt (due to ERS), and subjects perform the motor task around the middle of the given time. Thereby, the visual stimulation provoked by changes of images in the screen that affect the EEG signals are avoided. If a movement is not performed in this time interval, the trial is discarded. Consequently, around 360 s of data are obtained for the relax and for elbow extension/flexion periods.

In this first phase, the orthosis supports the arm but FES is deactivated. Instead, the researcher moves the subject arm when the dot in the screen disappears (and the subject is doing a motor task) and also helps the subject to keep the arm flexed or extended if necessary. Thus, the subject can get relaxed more easily.

The second phase of the experiment consists in performing the same tasks, but testing the SVM classifier in real-time. Then, FES is activated when the classifier detects the intention to perform an elbow flexion/extension movement (unless it is not needed, i.e. the user is able to perform it on their own) and the experimenter only helps to keep the arm flexed or extended. In this phase, four runs are performed. As in the previous experiment, five extra seconds are established to allow the FES control and to avoid spurious data. This extra time is included when the dot in the screen disappears.

The order of the tasks depends on the behavior of the BMI system itself. When the classifier detects correctly the task that the subject is performing, the screen shows the next task following the same order that was used in the first phase (rest state, elbow extension, rest state, elbow flexion and so on). If not, the screen shows the same task and the subject has to try again.

As before, the data used to test the SVM-based classifier are the four seconds in the middle of the dot period (when the subject is performing the task, labeled as “Used data” in Fig. 2b). The classifier decision is taken when the dot disappears.

#### Signal processing

As previously stated, 16 EEG electrodes distributed around parietal and frontal lobes are used to register the brain activity. To achieve suitable features to distinguish between movement intention and rest, the considered data (“Used data” in Fig. 2b) are processed in the same way as in the previous approach (bandpass filter and Laplacian algorithm).

The next step consists of transforming the time domain of the EEG signals into the frequency domain and using the PSD of mu and beta frequency bands as features. Therefore, the Fast Fourier Transform (FFT) is applied and the sums of three relevant PSD bands per each electrode are used as features. To get these features, mu and beta frequencies are divided into the band components normally involved in ERD which are 8–12 Hz (mu frequencies) and 13–24 Hz (low beta frequency band), and into the band involved in ERS (25–30 Hz, high beta band components). The sums of these three bands are used to classify the different tasks performed.

#### Classification

An analogous SVM-based classifier mentioned earlier is used in this experiment. The classifier is trained in order to predict if the subject is resting or trying to perform an elbow flexion/extension movement. With regard to the classifier, both movements are considered as the same class, so the position of the arm is used to differentiate these two states.

In the first phase of the experiment, each subject performs an offline test whose data are used to train seven models of the classifier using all possible combinations of the three features extracted per electrode (see Table 2). These combinations allow the selection of seven different options related to mu, low beta and high beta frequency bands. Then, the best one in terms of accuracy is used in the second phase where the data are processed in real-time.

**Table 2:** Combinations of features to train the SVM models

	Combination						
	1	2	3	4	5	6	7
8–12 Hz	x			x	x		x
13–24 Hz		x		x		x	x
25–30 Hz			x		x	x	x

Columns represent the combination number assigned and rows are the sums of frequencies (features). An X indicates which frequency bands are used in each combination

## Results and discussion

### Motor imagery results

First, an initial training is necessary to generate a model which supports the SVM-based classifier to detect the motor imagery tasks. As mentioned, four training runs were performed, considering a variable length of the rest state trials. The recorded signals during this offline phase were analyzed taking into account three different lengths. This analysis with imbalance data was performed to optimize the detection of the mental tasks and to reduce the false detection of motor imagery tasks. Due to the amount of time needed to take the test, patient P4 felt fatigued and was not able to finish it.

The accuracy of the system was checked using a 4-fold cross-validation, where each run acts as a fold. The accuracy (ACC) of the generated models and the selected length of trials for each user (marked in bold) are shown in Table 3. The different lengths of trials were selected in proportion to the length of the trials for the motor imagery task (MIT). These lengths were selected as 1:1, 2:1 or 3:1 (rest state:motor imagery). In addition to the overall accuracy of the model, accuracy in the differentiation of each task is individually shown. The proportion of length between trials was selected individually taken into account not only the total accuracy but also the reduction of False Positives (maximizing the accuracy of the rest state detection). All the cases (except for user P2 who used 3:1 data) used the relation 2:1 for the data. The average accuracy of the selected options for healthy users, patients and both is also shown. These average values show similar accuracy in the detection of the rest state (87.8 % for healthy users and 85.3 % for patients). However, the accuracy for the MIT is better for the healthy subjects (45.9 %) than for the patients (36.9 %).

**Table 3:** 4-fold cross-validation results of the MIT offline tests

Volunteer	MIT ACC (%)	Relax ACC (%)	Total ACC (%)
H1 - 1:1	52.3 ± 8.4	56.3 ± 5.6	54.3 ± 2.4
**H1 - 2:1**	**12.2 ± 8.4**	**87.5 ± 6.0**	**49.8 ± 3.7**
H1 - 3:1	0.3 ± 0.6	99.5 ± 0.8	49.9 ± 0.6
H2 - 1:1	89.1 ± 6.0	85.9 ± 9.4	87.5 ± 4.8
**H2 - 2:1**	**83.2 ± 11.3**	**92.8 ± 6.2**	**88.0 ± 5.5**
H2 - 3:1	79.3 ± 13.7	95.7 ± 3.7	87.5 ± 6.3
H3 - 1:1	71.7 ± 13.3	60.2 ± 19.7	65.9 ± 5.8
**H3 - 2:1**	**42.4 ± 15.5**	**83.2 ± 12.3**	**62.9 ± 1.9**
H3 - 3:1	33.8 ± 14.9	91.7 ± 6.5	62.8 ± 5.4
Healthy users average	**45.9 ± 35.6**	**87.8 ± 4.8**	**66.9 ± 19.4**
P1 - 1:1	67.1 ± 24.9	32.9 ± 28.1	57.1 ± 1.7
**P1 - 2:1**	**40.8 ± 29.0**	**74.8 ± 23.4**	**57.8 ± 9.9**
P1 - 3:1	8.2 ± 12.3	95.0 ± 8.8	51.6 ± 2.0
P2 - 1:1	66.1 ± 13.4	65.5 ± 10.8	65.8 ± 2.5
P2 - 2:1	43.8 ± 13.6	88.2 ± 4.2	66.0 ± 4.8
**P2 - 3:1**	**32.2 ± 12.8**	**94.9 ± 2.8**	**66.0 ± 5.4**
P3 - 1:1	76.3 ± 9.2	72.7 ± 18.8	74.5 ± 8.6
**P3 - 2:1**	**55.6 ± 9.5**	**84.4 ± 10.5**	**70.0 ± 4.4**
P3 - 3:1	44.4 ± 7.3	89.2 ± 9.0	66.8 ± 1.9
P5 - 1:1	61.2 ± 6.5	49.7 ± 9.8	55.4 ± 4.4
**P5 - 2:1**	**19.1 ± 5.1**	**87.0 ± 3.9**	**53.0 ± 2.3**
P5 - 3:1	8.6 ± 1.7	97.5 ± 0.9	53.0 ± 0.7
Patients average	**36.9 ± 15.3**	**85.3 ± 8.3**	**61.7 ± 7.7**
Total average	**40.8 ± 23.7**	**86.4 ± 6.6**	**63.9 ± 12.8**

Healthy users (H) and patients (P)

After the creation of the classifier models and their analysis, we could realize that the users, generally, did not have a model which was able to differentiate clearly between the two mental tasks. This behavior can complicate the control of the movements of the exoskeleton in real-time. However, the selection of the length of the rest state data was able to reduce the False Positives, allowing a better control of the system with the drawback of making it slower. By using these models, the users performed the experimental test described earlier. Tables 4 and 5 show three different parameters to define the behavior of the system. The True Positive Rate (TPR) is calculated as the percentage of motor imagery tasks detected correctly. On the other hand, the False Positive Rate (FPR) represents the percentage of motor imagery tasks detected in the rest periods. Finally, the accuracy of the system (ACC) is calculated as the percentage of right detection taking into account both motor imagery as well as rest state. It is necessary to state that false positives did not activate the FES since it was known which task the user should be performing every time. Therefore, resting periods detected as motor imagery tasks did not move the arm (they were only taken into account to calculate the FPR).

**Table 4:** Results of the motor imagery online tests. Healthy volunteers

Volunteer	Run	TPR (%)	FPR (%)	ACC (%)
H1	1	30	30	50
	2	90	20	85
	3	60	20	70
	4	50	20	65
	Average	57.54 ± 25	22.5 ± 5	67.5 ± 14.4
H2	1	100	0	100
	2	100	10	95
	3	100	10	95
	4	100	10	95
	Average	100 ± 0.0	7.5 ± 5.0	96.25 ± 2.5
H3	1	90	40	75
	2	100	20	90
	3	100	20	90
	4	100	30	85
	Average	97.5 ± 5.0	27.5 ± 9.6	85.0 ± 7.1
Total average		85.0 ± 24.3	19.2 ± 10.8	82.9 ± 15.0

**Table 5:** Results of the motor imagery online tests. Patients

Volunteer	Run	TPR (%)	FPR (%)	ACC (%)
P1	1	40	10	65
	2	30	20	55
	3	50	10	70
	4	50	30	60
	Average	42.5 ± 9.6	17.5 ± 9.6	62.5 ± 6.5
P2	1	40	0	70
	2	40	10	65
	3	70	0	85
	4	50	10	70
	Average	50 ± 14.1	5.0 ± 5.8	72.5 ± 8.7
P3	1	90	10	90
	2	50	10	70
	3	50	10	70
	4	40	20	60
	Average	57.5 ± 22.2	12.5 ± 5.0	72.5 ± 12.6
P5	1	40	10	65
	2	50	20	65
	3	20	30	45
	4	20	40	40
	Average	32.5 ± 15.0	25.0 ± 12.9	53.8 ± 13.1
Average		45.6 ± 17.1	15.0 ± 10.9	65.3 ± 12.4

**Fig. 3:**
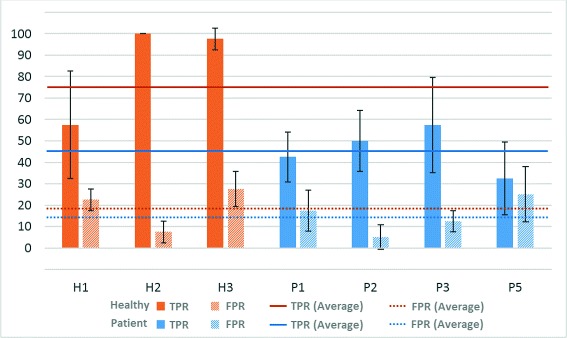
Motor imagery results - Online test. Percentages of TPR and FPR (and their average value) for healthy volunteers (H) and patients (P)

In the case of ACC, the behavior of the system was clearly better for healthy subjects (with an average of 82.9 ± 15.0 %) than for patients (65.3 ± 12.4 %). However, the FPR is similar for patients (15.0 ± 10.9 %) and healthy people (19.2 ± 10.8 %). Figure 3 shows the TPR and FPR values graphically for each user and their standard deviation and average (for healthy users and patients separately).

The system had a proper performance for healthy users (especially for users H2 and H3). In the case of patients, the results were more diverse. The system was able to detect around 50 % of the motor imagery task for patients P2 and P3, triggering the FES system and moving their affected arms with a reduced FPR (5 % and 12.5 %, respectively). However, patients P1 and P5 did not reach these TPRs and the FPR obtained was also higher.

### Movement intention results

**Fig. 4:**
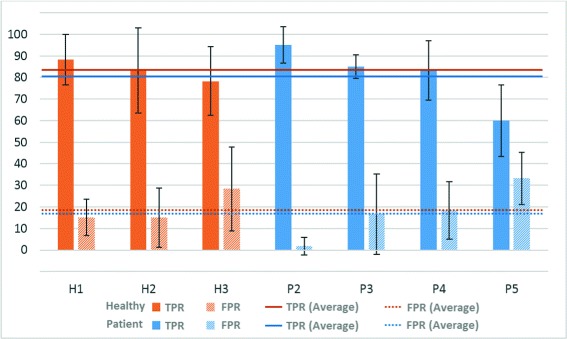
Movement intention results - Offline test. Percentages of TPR and FPR (and their average value) for healthy volunteers (H) and patients (P)

In order to estimate the performance of the predictive SVM-based models, a statistical analysis was done in the first phase of the experiment using a 6-fold cross-validation (where each fold is a run). This analysis was done for each combination of features and then, the best one was selected to be used in the second phase of the experiment. This analysis provides the accuracy of the system (ACC), the True Positive Rate (TPR) and the False Positive Rate (FPR). Figure 4 shows these values and their average. As in the previous test, one user (P1 in this case) was not able to finish this experiment due to fatigue. In addition to these values, in Table 6 the combination which provides the best results after processing offline the test data for both healthy and patient subjects is shown.

**Table 6:** 6-fold cross-validation results of the best combination of features for movement intention test

Volunteer	TPR (%)	FPR (%)	ACC (%)	Combination
H1	88.3 ± 11.7	15.0 ± 8.4	86.7 ± 10.1	7
H2	83.3 ± 19.7	15.0 ± 13.8	84.2 ± 16.8	5
H3	78.3 ± 16.0	28.3 ± 19.4	75.0 ± 17.7	2
Average	83.3 ± 15.7	19.4 ± 15.1	81.9 ± 15.4	-
P2	95.0 ± 8.4	1.7 ± 4.1	96.7 ± 6.3	7
P3	85.0 ± 5.5	16.7 ± 18.6	84.2 ± 12.1	7
P4	83.3 ± 13.7	18.3 ± 13.3	82.5 ± 13.5	7
P5	60.0 ± 16.7	33.3 ± 12.1	63.4 ± 14.4	1
Average	80.8 ± 17.2	17.5 ± 16.8	81.7 ± 17.0	-

Healthy users (H) and patients (P)

According to the results obtained in the offline analysis, all subjects seemed to be able to control the activation of the FES system using the BMI. However, P5 would find a big challenge to control the system appropriately in the online test due to the low rate of movement intentions correctly detected versus the high rate of resting time periods detected as movement intention (False Positive). This patient had special conditions which could distort the behavior of the system (see Section *namerefdiff*). Moreover, he had his best model using only mu frequencies, which was remarkably different from the other patients. It was expected that the remaining subjects (patients and healthy subjects) could successfully control the BMI system.

Combination 7 predominates over the rest of combinations (four out of seven) as all frequency bands normally involved in ERD and ERS are used. However, subjects P5, H2 and H3 achieved their best results discarding some bands. This might be because not everybody modulates in the same way their brain waves and it is necessary to search the best ones in order to manage an ERD/ERS-based system. However, the short period of time to conduct the experiment with patients made difficult the exhaustive searching for the best frequencies and this issue was simplified to the seven possible combinations described before.

In average, users were able to achieve an accuracy of 81.9 % and 81.7 % (healthy users and patients, respectively), 83.3 % and 80.8 % of TPR and 19.4 % and 17.5 % of FPR which are satisfactory values to control the BMI system. These values of TPR means that, more or less, eight out of ten times the user performed a task that the system was able to detect correctly. The models of the classifier obtained in this first phase of the experiment (offline) were used to control the system in the second phase (online).

**Fig. 5:**
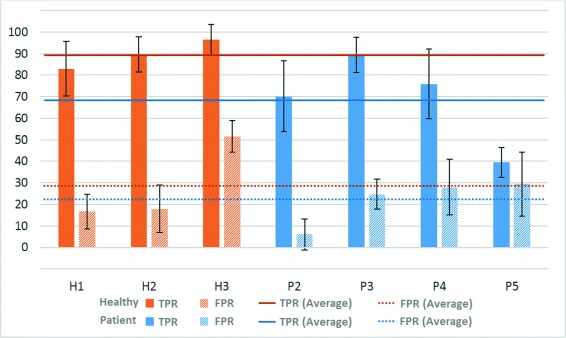
Movement intention results - Online test. Percentages of TPR and FPR (and their average value) for healthy volunteers (H) and patients (P)

Regarding the second part of this experiment, Tables 7, 8, 9 and 10 and Fig. 5 show the online results of healthy and patient subjects. Tables 8 and 10 add a system accuracy column (ACC). This value shows how many tasks were correctly detected in relation to the total number of tasks performed (in percentage). As it was mentioned before, each task detected wrongly had to be performed again until it was correctly detected. Therefore, the sequence and the number of repetitions of each task is variable. However, in the end, the users had to perform twenty tasks per run. If the system worked perfectly, the sequence of tasks remained as in the offline tests. Thus, the users had feedback about how they were doing the task and how they could adapt their concentration to the task. As in the prior method, a wrong detection of the movement intention did not provoke an activation of the FES system.

**Table 7:** Healthy subjects results in online movement intention test

Volunteer	Run	Rest task		Extension task		Flexion task	
		R	W	R	W	R	W
H1	1	8	1	4	2	3	2
	2	9	1	5	1	5	0
	3	8	3	4	0	4	1
	4	9	2	4	0	4	1
	Average	8.5 ± 0.6	1.8 ± 1.0	4.3 ± 0.5	0.8 ± 1.0	3.8 ± 0.5	1.0 ± 0.8
H2	1	8	4	4	0	4	0
	2	9	1	5	1	4	0
	3	9	2	4	1	4	0
	4	9	1	4	0	4	2
	Average	8.8 ± 0.5	2.0 ± 1.4	4.3 ± 0.5	0.5 ± 0.6	4.0 ± 0.0	0.5 ± 1.0
H3	1	5	10	3	0	2	0
	2	7	6	3	0	3	1
	3	6	9	3	0	2	0
	4	8	4	4	0	4	0
	Average	6.5 ± 1.3	7.3 ± 2.8	3.3 ± 0.5	0.0 ± 0.0	2.8 ± 1.0	0.3 ± 0.5
Total average		7.9 ± 1.3	3.7 ± 3.1	3.9 ± 0.7	0.4 ± 0.7	3.4 ± 0.8	0.6 ± 0.8

R (Right) and W (Wrong) columns of each task determine how many tasks were detected correctly or not, respectively

**Table 8:** Healthy subjects results in online movement intention test. Accuracy of the system

Volunteer	Run	TPR (%)	FPR (%)	ACC (%)
H1	1	63.6	11.1	75.0
	2	90.0	10.0	90.0
	3	88.9	27.3	80.0
	4	88.9	18.2	85.0
	Average	82.9 ± 12.8	16.6 ± 8.0	82.5 ± 6.5
H2	1	100.0	33.3	80.0
	2	90.0	10.0	90.0
	3	88.9	18.2	85.0
	4	80.0	10.0	85.0
	Average	89.7 ± 8.2	17.9 ± 11.0	85.0 ± 4.1
H3	1	100.0	66.7	50.0
	2	85.7	46.2	65.0
	3	100.0	60.0	55.0
	4	100.0	33.3	80.0
	Average	96.4 ± 7.2	51.5 ± 14.9	62.5 ± 13.2
Total average		89.7 ± 10.5	28.7 ± 19.9	76.7 ± 13.2

**Table 9:** Patient results in online movement intention test

Volunteer	Run	Rest task		Extension task		Flexion task	
		R	W	R	W	R	W
P2	1	6	1	3	0	3	7
	2	9	0	5	1	4	1
	3	9	0	4	0	4	3
	4	9	1	4	0	4	2
	Average	8.3 ± 1.5	0.5 ± 0.6	4.0 ± 0.8	0.3 ± 0.5	3.8 ± 0.5	3.3 ± 2.6
P3	1	9	2	4	0	4	1
	2	8	4	4	0	4	0
	3	8	3	4	0	4	1
	4	8	2	4	2	4	0
	Average	8.3 ± 0.5	2.8 ± 1.0	4.0 ± 0.0	0.5 ± 1.0	4.0 ± 0.0	0.5 ± 0.6
P4	1	7	3	4	3	3	0
	2	7	4	3	3	3	0
	3	7	2	4	4	3	0
	4	10	1	5	0	4	0
	Average	7.8 ± 1.5	2.3 ± 1.3	4.0 ± 0.8	2.5 ± 1.7	3.0 ± 0.5	0.0 ± 0.0
P5	1	5	1	2	8	3	1
	2	5	4	2	4	2	3
	3	6	4	3	2	3	2
	4	5	1	3	6	2	3
	Average	5.3 ± 0.5	2.5 ± 1.7	2.5 ± 0.6	5.0 ± 2.6	2.5 ± 0.6	2.3 ± 1.0
Average		7.4 ± 1.6	2.1 ± 1.4	3.6 ± 0.9	2.1 ± 2.5	3.4 ± 0.7	1.5 ± 1.9

Columns R (Right) and W (Wrong) of each task determine how many tasks were detected correctly or not, respectively

**Table 10:** Patient results in online movement intention test. Accuracy of the system

Volunteer	Run	TPR (%)	FPR (%)	ACC (%)
P2	1	46.2	14.3	60.0
	2	81.8	0.0	90.0
	3	72.7	0.0	85.0
	4	80.0	10.0	85.0
	Average	70.2 ± 16.5	6.1 ± 7.2	80.0 ± 13.5
P3	1	88.9	18.2	85.0
	2	100.0	33.3	80.0
	3	88.9	27.3	80.0
	4	80.0	20.0	80.0
	Average	89.5 ± 8.2	24.7 ± 7.0	81.3 ± 2.5
P4	1	70.0	30.0	70.0
	2	66.7	36.4	65.0
	3	66.7	36.4	65.0
	4	100.0	9.1	95.0
	Average	75.1 ± 16.8	24.4 ± 11.7	75.0 ± 13.5
P5	1	35.7	16.7	50.0
	2	36.4	44.4	45.0
	3	50.0	40.0	55.0
	4	35.7	16.7	50.0
	Average	39.5 ± 7.0	29.5 ± 14.9	50.0 ± 4.1
Average		68.5 ± 22.1	21.2 ± 13.3	71.6 ± 15.8

In general, users achieved a satisfactory level of control (in average, TPR = 77.6 ± 20.7 %, FPR = 24.4 ± 16.6 % and ACC = 73.8 ± 14.7 %), although they needed one or more runs to get used to the system since the electrical stimulation was somewhat unexpected for them and could be distracting. Moreover, sometimes they got frustrated if they did not activate the FES system when they had tried a movement.

Healthy users controlled satisfactorily the system. H1 and H2 had more ability to control the BMI system than H3. As offline and online results showed, H3 had more difficulties at keeping at rest. For this user, in our opinion, the false positive and accuracy rates obtained were not good enough to be successful. Perhaps, it could be interesting to obtain an personalized features extraction of the sensorimotor rhythms for this specific user. Moreover, the BMI system used with H3 only used low beta frequencies, so the ERS phenomenon was not analyzed. On the other hand, H1 and H2 reached 90 % of ACC which is a desirable level. For all healthy users, the resting task was more challenging than the motor tasks in view of the number of wrong tasks counted.

In view of the results of patients who have suffered a stroke with hemiplegia (P2, P3 and P4), it is possible to state that an ERD/ERS-based system could be used in the rehabilitation process since they achieved around 78.75 % of ACC. However, the FPR was 18.40 % and it should be reduced to zero in order to avoid a malfunctioning of the system, provoking undesirable arm movements. Compared with offline tests, the results have only gotten worse slightly (around 88 % of ACC), which shows the stability and reliability of the system.

In the case of subject P5, he was not able to control the system. Probably, with more time to explain and perform the experiment, he would had achieved better results. On the other hand, subject P4 had more difficulties with the resting and extension tasks, subject P3 with the resting task and user P2 with the flexion task. But in all cases, they only needed a few attempts to go on with the sequence of tasks.

By comparison with healthy users, the BMI system used with patients had more problems to detect the movement intentions. This could be due to the fact that the ERD and ERS phenomena diminish progressively after the stroke.

### Comparison of methods

Both methods presented in this work show similar behavior for healthy subjects and patients. In terms of accuracy, both methods had similar values. Healthy subjects were able to obtain, in average, an accuracy of 82.9 % and 76.7 % (for motor imagery and movement intention detection, respectively). Regarding the TPR, we noticed similar values for healthy users (85.0 % in the motor imagery task and 89.7 % in the movement intention detection). It is worth to mention that the FPR was slightly better in the motor imagery tests (19.2 %) than in the movement intention ones (28.7 %).

In relation to results with patients, although the global accuracies were similar (65.3 % for motor imagery and 71.6 % for movement intention detection), both TPR and FPR showed important differences. The motor imagery method in patients presented a lower TPR (45.6 % against the 68.5 % obtained in movement intention detection) but the FPR was better (15.0 % and 21.2 %, respectively).

It should be considered that all users are BMI-naive and a long training period is usually needed to learn to modulate the brain potentials (this fact is particularly relevant in motor imagery tasks). Moreover, the system was successfully validated in previous studies [23, 29] but always with healthy subjects. In this study, we want to demonstrate the feasibility of the system in patients, rather than its final design. For better classifications, we should make a more detailed analysis of EEG signals from each user (due to different neurological conditions). This way, we could customize the BMI system, focusing the electrodes on the patients’ brain areas with more activity during the performance of the required tasks.

Depending on the target of the real-time application, it could be more interesting to use the motor imagery method (reducing the number of wrong detections) or the movement intention detection method (improving the rate of correct detections). If the number of wrong detections (i.e. the FPR) would be reduced, the subjects would not need the user interface shown in the computer to give them the instructions to control the exoskeleton. However, reducing FPR is really complex due to the variability of the EEG signals among people and inter-individual. Anyway, the second method works better for patients.

### Difficulties related to the patients

The test protocol was slightly different between healthy users and patients because patients usually had difficulties to perform the arm movements. All of them needed some help to keep the arm outstretched after an extension movement, although some patients (P2 and P3) could do the elbow flexion movement relatively easy. For these reasons, an experimenter helped the patients to complete the arm movements (flexion and/or extension) and to keep the arm immobilized in the rest periods.

P5 found extremely difficult to control appropriately the system due to his low rate of movement intentions correctly detected versus his high rate of resting time periods detected as movement intentions (FPs). This patient had suffered a brain injury that affected both cerebral hemispheres and speech. In addition, he manifested difficulty to focus on the experiment.

Some of the patients who have suffered a stroke with hemiplegia (P2, P3 and P4) were pleased to control at least the activation of their injured arm with their brain recovering some mobility. Moreover, they realized that they kept working some brain potentials related to the motor control of their paralyzed limb.

One of the concerns during the experiments was that stroke patients shrugged their shoulders or made a postural shift when they were demanded to move their arm, provoking EMG artifacts that diminish the quality of the EEG signals. In order to detect this kind of artifacts, the signals were visually inspected to detect outliers. The data acquired during the tests did not show any abnormal behavior and no significant outliers were detected.

The waveform produced during the ERD/ERS phenomenon (due to movement intention) is very difficult to detect. A single trial analysis has been performed to detect the phenomenon but this waveform is not clearly produced. For this reason, most of the authors show this phenomenon using averaged data during several movements (as in [50–53]). An analysis with averaged data using several trials has also been performed but, unfortunately, although the windows where the movements are performed are limited by the test, it is impossible to know the exact time when the users start the movement (the movements were self-paced and no system was used to know the particular time they were produced).

Anyway, before performing the experiments with the stroke patients, both BMI systems (using imagined and attempted movements) were tested with healthy users [23, 47, 54, 55] and it was verified that they did not move any part of their bodies when they were using the BMI. Thus, artifacts were not produced and only EEG signals were used to control the systems. The similar results obtained in these tests suggest that the data classified both for healthy users and for patients were only related to the brain information (and not related to artifacts).

## Conclusions

In this work, a system based on a hybrid exoskeleton for the upper limb rehabilitation of the patients with a neurological condition has been developed and tested. The hybrid system is composed of a passive exoskeleton to counteract the gravity effects and a FES system to drive the elbow flexion/extension movement. A BMI based in two different methods has been used to command the execution of this movement: one method uses motor imagery, and the other one detects the intention of movement.

Even though the accuracy of the system for some users seems to not be high enough, most of them were able to command the system by the BMI using both methods, being able to perform the whole test. Thus, it has been proved that this system could be applied for rehabilitation of the upper limb, including an active involvement of the patients in this process. As it was mentioned before, in the final application for patients’ rehabilitation, the movement intention detection method seems to be more appropriate than the motor imagery approach. On the other hand, two patients were not able to finish some tests because of the fatigue (not due to a malfunction of the system). For this reason, a shorter training should be designed to avoid this circumstance.

In future works, clinical trials must be performed in long-term therapies in order to verify if there is an improvement in the patients’ rehabilitation when this system is used. Related to the system behavior, the control strategy of the FES could be improved, making it adaptable to the residual motor capabilities of the patients. Moreover, other systems such as an active exoskeleton can be used to help the patient in the execution of the arm movements, avoiding the possible discomfort that the FES can cause to the users.

## Abbreviations

BMI: Brain-machine interface
FES: Functional electrical stimulation
CVA: Cerebrovascular accident
FP: False positive
EMG: Electromyography
EEG: Electroencephalography
ERD: Event-related desynchronization
ERS: Event-related synchronization
API: Application programming interface
PID: Proportional-integral-derivative
PSD: Power spectral density
DFT: Discrete fourier transform
SVM: Support vector machine
RBF: Radial base function
FFT: Fast fourier transform
ACC: Accuracy
MIT: Motor imagery task
TPR: True positive rate
FPR: False positive rate
